# Engineered ferritin for lanthanide binding

**DOI:** 10.1371/journal.pone.0201859

**Published:** 2018-08-13

**Authors:** Lorenzo Calisti, Matilde Cardoso Trabuco, Alberto Boffi, Claudia Testi, Linda Celeste Montemiglio, Amédée des Georges, Irene Benni, Andrea Ilari, Bartłomiej Taciak, Maciej Białasek, Tomasz Rygiel, Magdalena Król, Paola Baiocco, Alessandra Bonamore

**Affiliations:** 1 Department of Biochemical Sciences "Alessandro Rossi Fanelli", Sapienza University of Rome, Rome Italy; 2 Molirom srl, Rome, Italy; 3 Center for Life Nano Science @ Sapienza, Istituto Italiano di Tecnologia, Rome, Italy; 4 Institute of Molecular Biology and Pathology, National Research Council, Rome, Italy; 5 The City University of New York Advanced Science Research Center, New York, NY; 6 Faculty of Veterinary Medicine, Warsaw University of Life Sciences, ul. Nowoursynowska, Warszawa, Poland; 7 Cellis Ltd., Warsaw, Poland; 8 Department of Immunology, Center for Biostructure Research, Medical University of Warsaw, Warsaw, Poland; Russian Academy of Medical Sciences, RUSSIAN FEDERATION

## Abstract

Ferritin H-homopolymers have been extensively used as nanocarriers for diverse applications in the targeted delivery of drugs and imaging agents, due to their unique ability to bind the transferrin receptor (CD71), highly overexpressed in most tumor cells. In order to incorporate novel fluorescence imaging properties, we have fused a lanthanide binding tag (LBT) to the C-terminal end of mouse H-chain ferritin, HFt. The HFt-LBT possesses one high affinity Terbium binding site per each of the 24 subunits provided by six coordinating aminoacid side chains and a tryptophan residue in its close proximity and is thus endowed with strong FRET sensitization properties. Accordingly, the characteristic Terbium emission band at 544 nm for the HFt-LBT Tb(III) complex was detectable upon excitation of the tag enclosed at two order of magnitude higher intensity with respect to the wtHFt protein. X-ray data at 2.9 Å and cryo-EM at 7 Å resolution demonstrated that HFt-LBT is correctly assembled as a 24-mer both in crystal and in solution. On the basis of the intrinsic Tb(III) binding properties of the wt protein, 32 additional Tb(III) binding sites, located within the natural iron binding sites of the protein, were identified besides the 24 Tb(III) ions coordinated to the LBTs. HFt-LBT Tb(III) was demonstrated to be actively uptaken by selected tumor cell lines by confocal microscopy and FACS analysis of their FITC derivatives, although direct fluorescence from Terbium emission could not be singled out with conventional, 295–375 nm, fluorescence excitation.

## Introduction

Ferritin is a cage-like protein made of 24 subunits arranged in octahedral 432 symmetry with an outer diameter of roughly 12 nm and an inner diameter of 8 nm [1,2]. The symmetrical positioning of three or four subunits in the protein shell results in the formation of eight channels connecting the inner cavity to the outside and allows for the entry and exit of iron and other cations with a relatively broad selectivity [3]. Besides their physiological function, centered around intracellular iron uptake, the nanocage properties of ferritins have been exploited in a number of diverse biotechnological applications as drug delivery vectors [4], scaffolds for vaccine development [5] and tools for bioimaging [6]. In this context, ferritins have been proven particularly useful for the selective targeting to cell populations overexpressing the transferrin receptor (CD71), in particular iron avid tumor cells [7,8]. Ferritin H-homopolymers are in fact endowed with unique properties that confer themselves several advantages over CD71 main ligand, *i*.*e*. transferrin, and even over targeted antibodies. First of all, ferritins display a 250 Å^3^ hollow cage capable of hosting tens to hundreds small molecules, either physically “encapsulated” or covalently attached to the inner surface of the cavity. In contrast, transferrin or conventional antibodies can be engineered to covalently attach only a few molecules in selected positions far from the receptor recognition epitopes. Thus, ferritins display a very convenient “drug/protein ratio” with respect to the classical “drug/antibody ratio”. As a second instance, it must be considered that ferritins display 24 symmetrically related recognition epitopes for the CD71 receptor instead of the two of classical antibodies or two of transferrins. Overall, the multiplicity of recognition epitopes in ferritins results in a high affinity towards the target receptor (a property often referred to as “multivalence effect” [9]), that, in the case of CD71, rivals with typical antibodies affinities [10].

Among the many ingenious ferritin-based constructs for bioimaging, only a few have been devoted to the creation of smart fluorescent probes, and these include quantum dots gold nanoparticles and fluorescent metal chelators [11–13]. However, advanced optical imaging techniques need an expanded color palette of bright fluorescent probes for biological visualization in order to enable real-time cellular imaging with high spatial resolution for close-up view into subcellular compartments and for providing key information on intracellular activities and macromolecular dynamics.

In this framework, fluorescent probes based on trivalent lanthanide ions are becoming widespread due to their unique photophysical properties [14–16]. Lanthanide f-orbitals with their high quantum yields are capable of efficiently radiating most of the absorbed energy, although their small absorption cross sections pose limits to their use. To improve the luminescent signals, small organic fluorophores that absorb in the UV region and transfer the absorbed light to the lanthanide atom are thus currently used in complex with the metal ion. Complexes of lanthanides are characterized by narrow band emission spectra, large Stokes shift (150–300 nm), and excited state lifetimes within the range of micro to milliseconds. By exploiting the microsecond fluorescence of lanthanides, time-resolved spectroscopy allows for the elimination of the short living background signals whose lifetimes are usually not more than 10–15 ns and the enhancement of the sensitivity for recording the delayed signal [17]. Moreover, the inherently low extinction coefficient of lanthanide ions, due to the forbidden character of the electronic transitions, can be overcome by Förster resonance energy transfer (FRET) from an appropriately placed (within 5–6 Ǻ distance) sensitizer-fluorophore onto the emitting level of the lanthanide, a phenomenon often referred to as “antenna effect” [18]. Among lanthanides, Tb(III) and Eu(III) are the most interesting due to their more intense microsecond fluorescence in the visible region [19, 20]. In a first approach, lanthanide fluorescent chelators were directly incorporated within the ferritin cavity using the “encapsulation” method, which entails pH induced ferritin dissociation with subsequent trapping of the payload [12]. However, at least in the case of fluorescent probes, this method leads to a random and inhomogeneous distribution of the small organic molecules within the inner cavity, resulting in possible aggregation-induced quenching effects and/or leakage outside the cavity through the iron channels. Among the various methods for the incorporation of lanthanide ions into biomolecules, a straightforward and generalizable approach has been proposed in recent years that integrates a lanthanide binding sequence as a protein co-expression tag *via* molecular biology strategies. On the basis of known properties of calcium binding loops, recent design and engineering studies have resulted in the development of short polypeptides comprising 20 or fewer encoded aminoacids that are capable of tight and selective binding to lanthanides. These peptide sequences, identified by Imperiali’s group as “lanthanide-binding tags” (LBT), show low-nanomolar affinities for the target ions and are selective for lanthanides over other common metal ions [21–24]. LBTs thus represent a most convenient option for lanthanide protein labeling in that they can be directly encoded within a recombinant protein expression construct. Improved LBTs sequences have been developed that entails genetic encoding of polypeptide sequences at the N-terminal or C-terminal of a specific carrier protein or even insertion into specific protein loops [25]. The probe nature of these protein tags has been demonstrated by their use for luminescence-based visualization on gels, as magnetic-field paramagnetic alignment agents in protein NMR experiments [26, 27] in fluorescence microscopy [28], and as partners in luminescence resonance energy transfer (LRET) studies [29].

Among the number of proteins physiologically involved in metal binding that can be used for Terbium binding, ferritins may appear as one of the most suitable. In fact, native apoferritins have been reported to naturally bind Tb(III) within the iron binding sites [30]. In particular, it was demonstrated that mammalian apoferritins could bind more than one Tb(III) ion per subunit, corresponding respectively to ferroxidase site, threefold channel and nucleation centre [31]. Moreover, upon excitation between 280–295 nm Tb(III) ferritin complex showed characteristic emission bands at 490 (^5^D_4_-^7^F_6_ Tb electronic transition) and at 544 nm (^5^D_4_-^7^F_5_ Tb electronic transition) due to a FRET sensitization effect provided by aromatic aminoacids [31b]. However, the distance between Terbium ions and aromatic moieties in native ferritin isoforms made FRET efficiency very poor and suboptimal for any type of fluorescence/luminescence based measurement. Moreover, the original Terbium binding sites of the wild type protein were shown to exhibit variable affinities for Tb(III), with estimated Kd ranging from 2 to 666 μM at pH 6.4 [31a].

In the present paper, a high Tb(III) affinity LBT sequence has been genetically fused at the C-terminal end of the heavy chain of mouse ferritin. The tag has been designed to be located inside the 250 Ǻ^3^ wide inner cavity such that the lanthanide ions diffusing through the surface pores could eventually bind to the LBT sequence. The construct would thus act both as carrier targeted to CD71 receptors and as a FRET sensitizer. Mouse ferritin was used in view of the identical sequence within the CD71 binding region as the human ferritin sequence [32] and because of obviously more favourable immunogenic profile for forthcoming in vivo study in mouse.

## Methods

### Protein design, expression and purification

A synthetic gene encoding for mouse H chain ferritin (HFt) fused with a lanthanide binding peptide (LBT) was designed, synthesized, and optimized for *Escherichia coli* codon usage by Geneart (Geneart AG). The last five C-terminal aminoacids of H chain ferritin were replaced by the GSG spacer sequence, followed by the LBT sequence YIDTNNDGWIEGDELLA [24]. As such, the LBT sequence is designed to be located within the inner cavity, as a prolongation of the inward directed C-terminal region (or E helix in ferritin secondary structure nomenclature). The resulting HFt-LBT construct, was subcloned into a pET22-b vector (Novagene) between NdeI/XhoI restriction sites. A stop codon was inserted before the His-tag region to avoid transcription of the unwanted tag.

HFt-LBT was overexpressed in *Escherichia coli* BL21 cells upon induction with 1 mM IPTG (Isopropyl-β-D-1-thiogalactopyranoside) at OD_600_ = 0.6. Cells were harvested by centrifugation 16 hours post induction at 37°C.

Cells harvested from 1 L culture were resuspended in 20 mM HEPES buffer, pH 7.5, containing 300 mM NaCl, 1 mM TCEP (tris(2-carboxiethyl)phosphine), and a cOmplete Mini Protease Inhibitor Cocktail Tablet (Roche). Cells were disrupted by sonication and the soluble fraction was purified by heat treatment at 78°C for 10 minutes. Denatured *E*. *coli* proteins were removed by centrifugation at 12000 rpm at 4°C for 1 hour. The soluble protein was further purified by ammonium sulfate precipitation. The precipitated fraction at 70% ammonium sulfate was resuspended in 20 mM HEPES, 150 mM NaCl, pH 7.5 and dialyzed versus the same buffer. As final purification step, the protein was loaded onto a HiLoad 26/600 Superdex 200 pg column previously equilibrated in the same buffer using an ÄKTA-Prime system (GE Healthcare) (S1 Fig). Purified protein (S2 Fig) was concentrated to obtain the final protein preparation of 1 mg/mL and protein concentration was calculated by measuring the UV spectrum using an extinction coefficient of 26000 M^-1^cm^-1^. A small amount of higher molecular weight aggregates was observed in all ferritins preparations (less than 5%), and attributed to the presence of intermolecular disulphide bridges, possibly involving the surface exposed Cys103 (S1 Fig and inset). As indicated Protein yield was ~50 mg/L culture.

The expected molecular weight of 22662 Da was confirmed by MALDI-TOF Mass Spectrometry as reported in Supplementary Data (S3 Fig).

### MALDI-TOF mass spectrometry

40 μl of protein sample were desalted on a C8 Empore Disk (3M, Minneapolis, MN) home-made stage tip and resuspended in 3 μl formic acid 1%. 1 μl was spotted on a MALDI sample plate and allowed to air dry. Recrystallized sinapinic acid (SA matrix from Thermo Fisher Scientific) was prepared at a concentration of 5 mg/ml in 50:50 acetonitrile/water (0.1% Formic Acid) and spotted directly prior to insertion into the mass spectrometer.

Matrix-assisted laser desorption ionization (MALDI) mass spectra were acquired on 4800 MALDI-TOF/ TOF mass spectrometer (Applied Biosystems, Foster City, CA) equipped with a nitrogen laser operated at 336 nm laser. Acquisitions were performed in linear mode averaging 2500 laser shots in a random, uniform pattern. Ions were accelerated with a 20 kV pulse, with a delayed extraction period of 860 ns. Spectra were generated by averaging between 500 and 2000 laser pulses in a mass range from 4 kDa to 50 kDa. Laser intensity was set to optimize the signal-to-noise ratio and the resolution of mass peaks of the analyte. All spectra were externally calibrated and processed via Data Explorer software (version 4.7).

### Fluorescence spectroscopy

Fluorescence spectra and titrations were performed using FluoroMax 4 (Horiba) spectrofluorimeter with a Haake D8 refrigerated bath at 20°C. Emission spectra were recorded between 450 and 560 nm, in order to include the luminescent maxima of Tb(III) (490 and 545 nm). The excitation wavelength was chosen at 295 nm to minimize the overlap of second order diffraction (570 nm) with the Tb(III) emission at 545 nm. Emission spectra were taken with excitation and emission band passes of 4 and 8 nm and corrected for the blank contribution and the instrument response at 295 nm in a quartz cell of 1 cm pathlength. Emission spectra were normalized to 1 at 545 nm.

Fluorescence static spectra were performed using 1 μM (monomer) apoHFt-LBT and wild type apoHFt as a control in 100 mM MES buffer pH 6.4. A 50 mM TbCl_3_ anhydrous powder (Sigma Aldrich) stock solution was also prepared in MES buffer at pH 6.4. Under these conditions, precipitation of Terbium hydroxides, easily formed around neutrality, is avoided. Fluorescence spectra of the protein Tb(III) complexes were recorded after 30 min incubation and after addition of 150 μM TbCl_3_ in buffer solution in order to saturate all possible Tb(III) binding sites both in HFt-LBT and HFt. Before recording spectra, protein solutions were exchanged with buffer (Terbium free) by centrifugal ultrafiltration on vivaspin MWCO 100 kDa concentrators (Sartorius) in order to remove unbound and weakly bound metal ions (5 exchange steps, 1×10 concentration each). Protein concentration was measured again and adjusted to the final concentration with buffer (1 μM monomer). Samples used for crystallographic and cryoEM measurements were also prepared according to this procedure.

Fluorescence titrations of HFt-LBT were carried out by adding incremental amounts (1.8 μL or multiples) of a 0.5 mM TbCl_3_ stock solution in 0.1 M MES buffer pH 6.4 to 3 mL of 1μM (monomer) protein in the same buffer under stirring. Emission spectra were recorded in 1 cm pathlength cuvette upon excitation at 295 nm at 25°C, 30 minutes after addition of TbCl_3_ solution aliquots. Fluorescence intensity of HFt-LBT Tb(III) complex as a function of the Tb(III)/HFt-LBT ratio has been reported. Fluorescent intensity was recorded at 545 nm corrected for the dilution factor and normalized to the emission maximum.

### Crystallization and X-ray structure determination

Crystals of wild type HFt-LBT and HFt-LBT in complex with Tb(III) were obtained by mixing in a 2 μl hanging drop the purified protein at 15 mg/ml with a solution containing 1.8/2.0 M ammonium sulphate and 0.1 M Tris, pH8.5, at 25° C within a week, cryo-protected by extensively washing the crystals in sodium malonate and flash-frozen in liquid nitrogen. Diffraction data were collected at λ = 1.0 Å and 1.4 Å respectively at XRD1 beamline at the Elettra Synchrotron, Trieste, Italy.

Data were processed with XDS [33] and scaled with Aimless [34] at final resolution of 2.85 Å and 2.65 Å, respectively. The structures were solved by Molecular Replacement with Phaser [35, 36] using the structure of mouse H-chain modified ferritin (pdb code 3WNW) without waters and ligands as a starting model. Model building and refinement were done using Coot [37] and Refmac5 [34]. Anomalous difference electron density map was calculated from the HFt-LBT Tb(III) crystal diffraction data, collected at the Tb emission peak (λ = 1.4 Å). The map has been generated using the Fast Fourier Transform Program belonging to the CCP4 suite [34]. The final model was analyzed with PROCHECK [38] and Molprobity [39]. Ramachandran Plot showed that more than 98% residues were in preferred regions and no outlier was observed in both structures. The validation of metal binding sites was performed using CheckMyMetal web server [40]. Final atomic coordinates and structure factors of apoHFt-LBT and HFt-LBT with Tb(III) were deposited in the PDB Data Bank (www.rcsb.org) with accession code 5OBA and 5OBB, respectively. Complete data collection and refinement statistics are reported in Supplementary Data (S1 Table) and validation reports were available for the review process.

### Cryo-electron microscopy

Holey-gold grids from Quantifoil R1.2/1.3 (Quantifoil Micro Tools GmbH) were prepared as described [41]. Grids surfaces were treated with plasma cleaning using a mixture of H_2_ and O_2_; then, 3 μl of a solution of 20 mM HEPES and 150 mM NaCl, pH 7.5, containing 1 μM (24-mer concentration) HFt-LBT Tb(III) complex was applied. After 30 s waiting time, grids were blotted (3 s) at 100% humidity with filter paper and vitrified by rapidly plunging into liquid ethane at −180°C with a Vitrobot Mark IV (FEI) [42, 43].

Data acquisition of 350 micrographs was performed using a FEI Titan Halo (FEI, Eindhoven) operating at 300 kV, while the specimen was maintained at liquid nitrogen temperatures. Data sets were collected with an automated data collection system [44] on a K2 Summit direct detector camera (Gatan, Pleasanton) operating in super-resolution mode, with a calibrated pixel size of 1.15 Å on the object scale and a magnification of 59000×. Images were typically recorded with a defocus range between −0.7 and −3.0 μm and using a dose of electrons on the specimen plane ranging between 10 and 20 electrons/Å^2^.

Data analysis was carried out using RELION 2.0 [45], while motion correction was performed using MotionCor2 [46]. A number of particles (91947) was picked and extracted from the original micrographs with the reference-based automated particle picking procedure implemented in RELION [44]; after the extraction, particles were 2D classified using 100 classes. Particles (48047) belonging to good 2D classes were selected and subjected to 3D classification, using as reference model the mouse H-ferritin structure (PDB code 3WNW), ultimately yielding 8 classes (S4 Fig). A selection of 35625 particles were subjected to another round of 3D classification. The resulting good class (7345 particles) was refined with the 3D Autorefine procedure in RELION and then subjected to the Post Processing step [47].

The final Post Processing 3D map resolution (equal to 7.1 Å) was estimated with the Fourier shell correlation (FSC) = 0.143 criterion, based on the ‘gold-standard’ protocol [48], using soft masks with a 4 pixel soft edge, and it has been corrected for the effects of the mask on the FSC curve using high-resolution noise substitution (S5 Fig) [49]. The final map was visualized using UCSF Chimera [50].

### Protein FITC labeling

HFt-LBT and HFt were labeled with Fluorescein-isothiocyanide (Sigma Aldrich) according to the manufacturer’s standard protocol. Briefly, 2 mg/ml (94 μM monomer) of purified protein was equilibrated in sodium carbonate buffer pH 9.0. 50 μl of 1 mg/ml (2.6 mM) FITC in DMSO were added to 1 ml of protein solution and the reaction mix was incubated under stirring for 2 hours at room temperature. The excess dye was removed by gel filtration chromatography and dye/protein ratio was determined by UV-spectroscopy. Both proteins were labeled with 0.95 FITC molecules per each subunit.

### Cell cultures and ferritins internalization

Human prostate cancer cell line DU-145 (ATCC HTB81), human colorectal cancer cell line HCT-116 (ATCC CCL-247), human breast cancer cell line MDA-MB-231 (ATCC HTB-26) and human ovarian cancer cell line SKOV-3 (ATCC HTB-77) were cultured in DMEM medium containing 10% FBS, 100 μg/ml streptomycin and 100 U/ml penicillin G in a humidified 37°C incubator. The internalization assay was performed as follow: cells were detached using trypsin, then washed twice with PBS and resuspended in non-supplemented DMEM medium containing FITC-ferritin nanoparticles (HFt-LBT or mouse HFt as a control) at the final concentration of 0.5 mg/ml for 1 hour in a humidified 37°C incubator. After incubation cells were washed three times with PBS and subjected for confocal microscopy and flow cytometry analysis.

### Confocal microscopy

Following internalization step described above, cells were seeded into 8-well Nunc Lab-Tek Chambered Coverglass with 200 μl DMEM medium containing 10% FBS, 100 μg/ml streptomycin and 100 U/ml penicillin G per well. Chambers with cells were then incubated on ice until microscopic visualization. Images were acquired using an inverted confocal microscope IX70 FV 500 (Olympus), with 488 nm laser, 20x objective lens and emission filter 505–560 nm. Image processing was performed using ImageJ software (National Institutes of Health, https://imagej.nih.gov/ij/) [51].

### Flow cytometry analysis

Cells were incubated with FITC-ferritin nanoparticles as described previously, then washed three times with PBS, and resuspended in FACS buffer (2% FBS, 1 mM EDTA). Internalization of ferritins before and after treatments was measured at the BD FACS Aria III equipped with a 488 nm laser. Cells were first gated by forward and side scatter area (FSC-A and SSC-A) plot and for singlets population (FSC-H and SSC-A), then detected in the channel for FITC expression (530/30nm filter) and side scatter parameter. The gate for the final detection was set according to the gate set on the control sample. Data were analyzed using BD FACSDIVA and FlowJo softwares.

## Results

### Fluorescence spectroscopy

**Fig 1:**
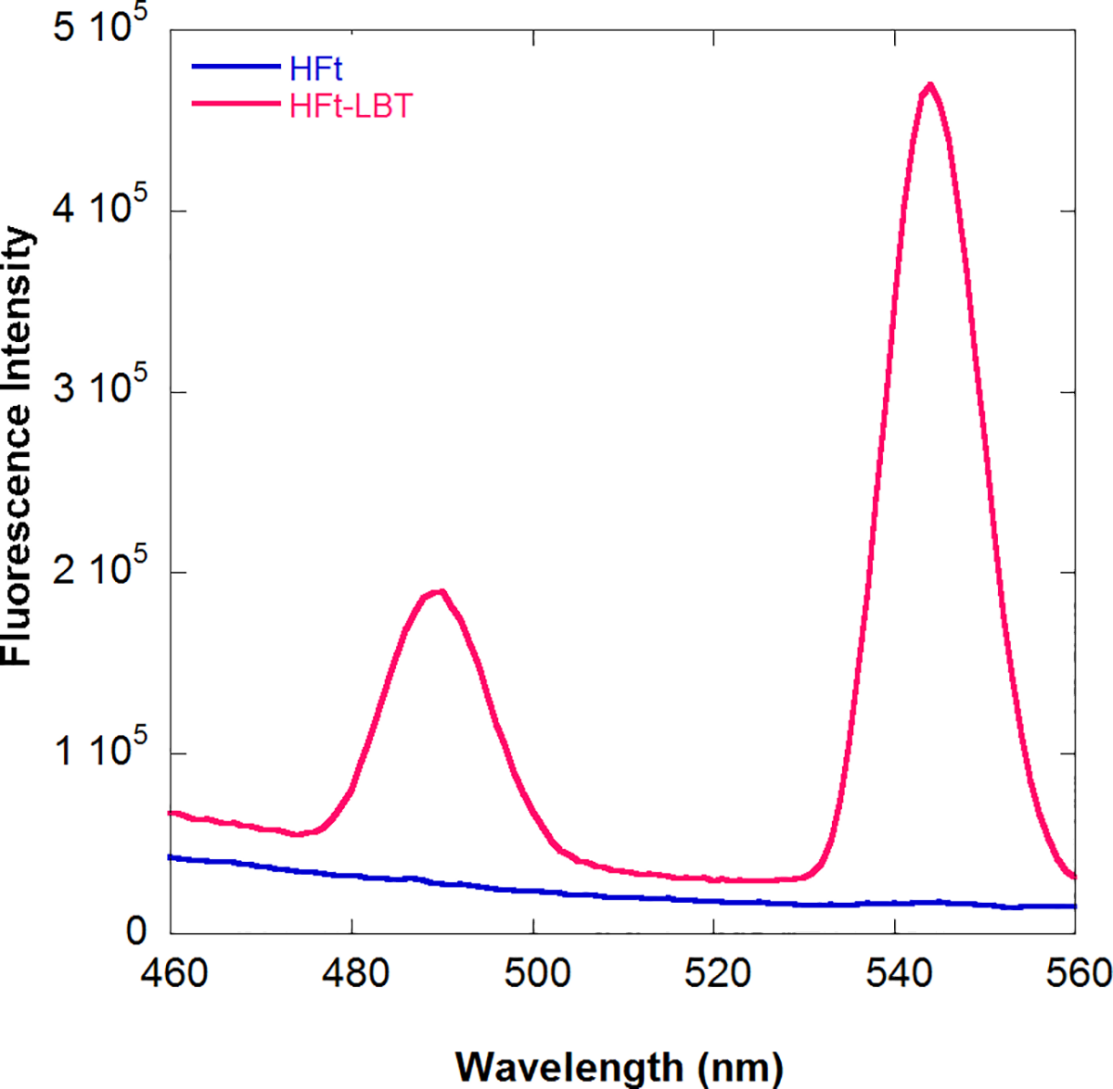
Fluorescence spectra of HFt-LBT Tb(III) complex. Fluorescence spectra of HFt-LBT Tb(III) (red line) and wild type mouse HFtTb(III) complexes (blue line) at the same protein concentration (1 μM monomer). The main emission band due to the ^5^D_4_-^7^F_5_ (Tb d-f orbitals) electronic transition at 544 nm is accompanied by the weaker ^5^D_4_-^7^F_6_ transition. Spectra were recorded after protein saturation with TbCl_3_ in 0.1 M MES buffer pH 6.4 (see Methods).

Static emission spectra were recorded for Tb(III) saturated HFt-LBT and wild type mouse HFt after removal of unbound Tb(III) by centrifugal ultrafiltration (see Methods, section 2.3), upon excitation at 295 nm. As reported in Fig 1, the intensity of the Terbium emission peak at 544 nm for HFt-LBT was at least two orders of magnitude higher with respect to HFt, indicating that the presence of the LBT sensitizes the Terbium signal due to the presence of a tryptophan residue within the tag at an efficient energy transfer distance in HFt-LBT.

**Fig 2:**
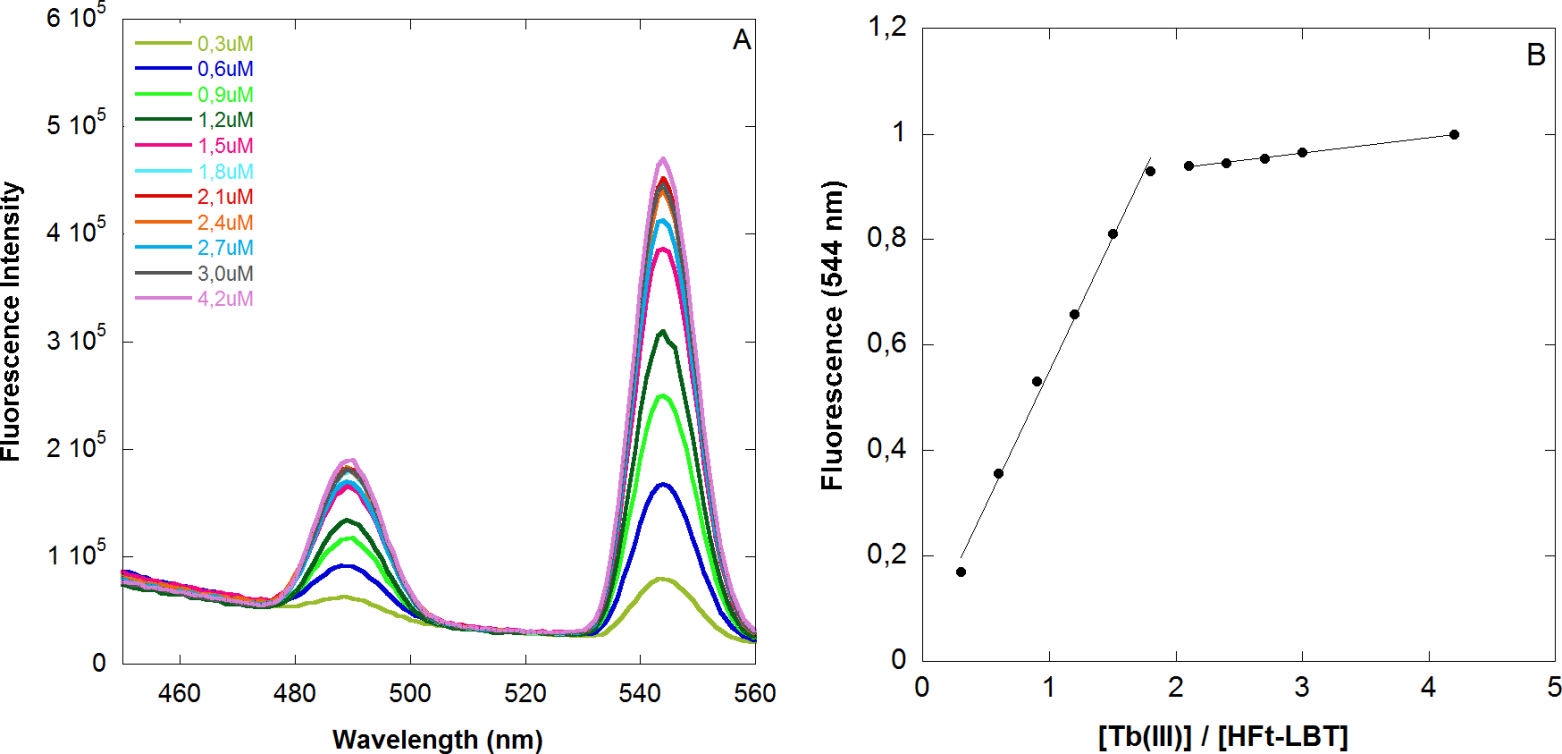
Fluorescence titration of HFt-LBT with Tb(III). A) Fluorescence titration of HFt-LBT (1μM monomer) with incremental concentration of Tb(III) in 0.1 M MES buffer pH 6.4. Emission spectra were recorded in 1 cm pathlength cuvette upon excitation at 295 nm. B) Fluorescence intensity of HFt-LBT Tb(III) complex as a function of the Tb(III)/HFt-LBT ratio. Fluorescent intensity was recorded at 545 nm, corrected for dilution factor and normalized to the emission maximum.

Fluorescence titration analysis was thus carried out on HFt-LBT by adding free Tb(III) ions to the apo protein (Fig 2A and 2B and Methods, section 2.3).

Intriguingly, the titration endpoint was reached at 1.7 equivalent amounts of Tb(III) per subunit instead of the predicted 1 equivalent based on the presence of one LBT moiety per monomer. Analogous titrations carried out on wild type mouse HFt, demonstrated a negligible fluorescence contribution provided by Terbium bound to the metal binding sites of the native protein (*i*.*e*. ferroxidase site, threefold channel and nucleation centre). Thus, the presence of the tryptophan residue within the LBT, appears to contribute for most of the observed fluorescence signal. The discrepancy between the observed and expected titration endpoint might consist in the additional contribution of the LBT tryptophan residue to the enhanced emission not only of the Tb(III) atom within the tag itself but also to other Tb(III) atoms present in the ferroxidase site or in the threefold channels. The demonstration of such effect may rely only on the generation of multiple mutants on the ferroxidase center and threefold channel that are unable to bind Tb(III). More interestingly, positioning of additional tryptophan residues next to these binding sites by mutagenesis could provide further enhancement of fluorescence/luminescence Tb(III) yields besides the LBT tag and they will be evaluated in the future. Further experimental work will also be needed in order to single out individual dissociation constants from each Tb(III) binding site both at equilibrium and in kinetic measurements. At present, the thermodynamics of this complex system can only rely on the published Kd relative to Tb(III) binding to the LBT of 57 nM [22, 23] and those relative to Tb(III) binding sites on wild type horse spleen heteropolymers, ranging from 2 to 666 μM [30]. Again, analysis of combined mutants will be necessary in order to solve the complex thermodynamics of the system. As a last comment, it is worth considering the possible effect of added iron II in solution on the stability of the HFt-LBT Tb(III) complex in view of the reported reversible competition among the diverse binding sites. A control experiment, depicted in S10 Fig, demonstrated that addition of stoichiometric amounts of iron II (1 iron atom per subunit) brought about a small decrease (2–3%) in the observed HFt-LBT Tb(III) fluorescent signal, accounting for possible displacement of Tb(III) ions from accessible sites, most likely the three-fold channels. No further decrease was observed over 48 h incubation and increasing concentration of added iron resulted in formation of insoluble iron oxide aggregates.

### HFt-LBT and HFt-LBT-Tb(III): Structural analysis

The structures of wild type apoHFt-LBT and HFt-LBT Tb(III) were determined by X-ray crystallography at 2.85 Å and 2.65 Å resolution, respectively. They both crystallized in I222 space group with 24 identical subunits in the asymmetric unit (ASU) with a solvent content of 64.7%. The overall X-ray-structures confirmed that the presence of the LBT tail does not affect the overall protein scaffold, which thoroughly matches the native H-chain mouse ferritin (pdb code 3WNW) with a *rmsd* value of 0.1 Å. However, the model building process allowed to position residues just from 3 to 176 since the LBT loop was not visible, probably because of the intrinsic high flexibility of the C-terminus region. When looking at the H-chain mouse ferritin structure, each ferroxidase center and 3-fold axes displayed one and two Mg(II) ions, respectively, due to the crystallization buffer solution. In a similar way, in each ferroxidase center and 3-fold axes of wild type apoHFt-LBT structure, a significant electron density peak was indeed observed and ascribed to iron ions (refined with occupancy <100%), since, although no salt was in the crystallization solution, the X-ray fluorescence scan displayed the presence of residual iron atoms. As a matter of fact, residual iron (5 atoms per ferritin 24-mer) is consistently present in recombinant ferritin H-homopolymers, most likely due to iron uptake within bacterial environment. In addition, in 3WNW structure, one iron ion was modelled in each 4-fold axis while in HFt-LBT structure, one water molecule was positioned and successfully refined.

When comparing the wild type apoHFt-LBT and HFt-LBT Tb(III), the LBT loop was still not visible while a very large electron density peak appeared in each ferroxidase center and in the eight 3-fold axes. In the X-ray emission scan, ranging from 4.0 to 21.0 KeV, the typical X-ray emission lines of Terbium were clearly identified in the HFt-LBT Tb(III) crystals (S6 Fig). Accordingly, the crystals of wild type apoHFt-LBT lacked these emission energies.

**Fig 3:**
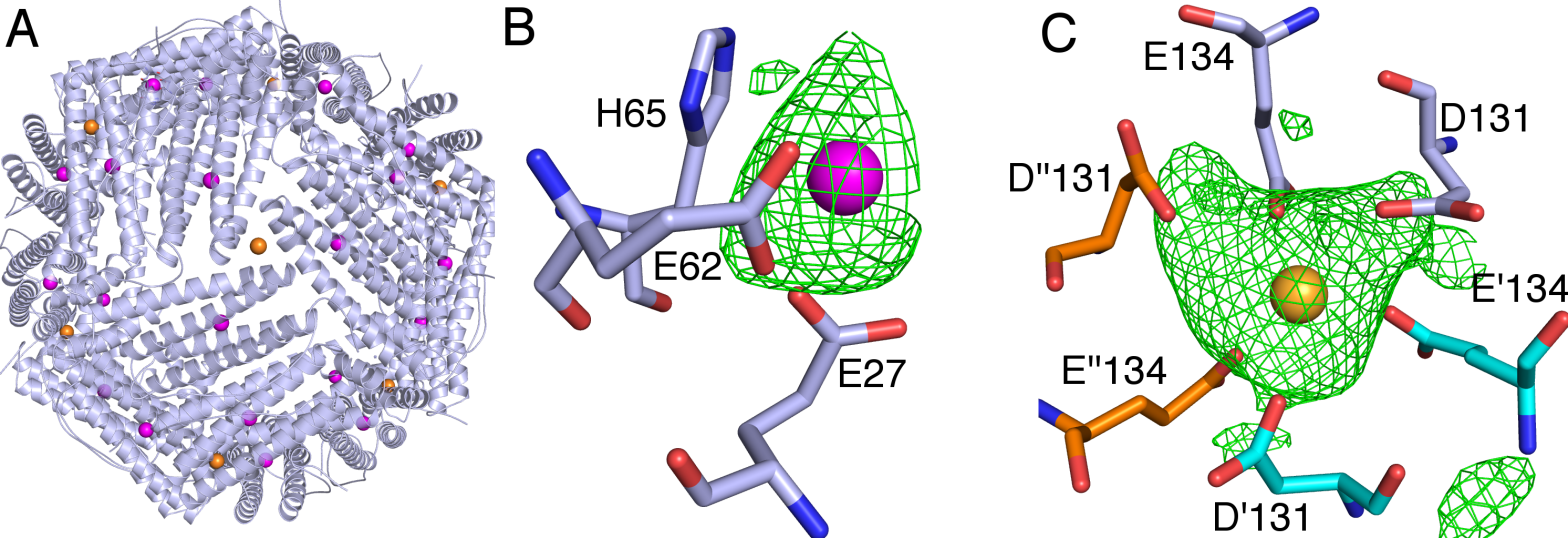
Terbium binding sites from X-ray crystallography. A) An overall view of the 24-meric shell of HFt-LBT Tb(III) showing the positions of Tb(III), displayed as spheres, is shown. In the close-up view, the omit map contoured at 3 σ as a green mesh is shown for the B) ferroxidase center where Tb(III) is depicted in magenta and for the C) 3-fold axes where Tb(III) is depicted as a yellow sphere and the residues Glu131 and Glu134 of three different monomers are depicted as sticks in light blue, cyan and orange, respectively.

As shown in Fig 3A, in HFt-LBT Tb(III) structure, 32 Terbium ions were positioned in each three-fold axis and in each ferroxidase site, depicted as yellow and magenta spheres, respectively. The position of Tb(III) ions was clearly assigned by comparison of the 2Fo-Fc electron density maps of the HFt-LBT Tb(III) and the wild type apoHFt-LBT was used as a control. An additional demonstration of the Terbium assignments was obtained by anomalous difference electron density map, which clearly displayed the positions attributed to Terbium (S7 Fig). In the ferroxidase center, each Terbium ion was successfully refined with 70–75% occupancy and was located in a trigonal planar coordination to OE1-Gln141 and to OE1 and OE2-Glu62, and to OE2-Glu27, in a range of 2.6–3.2 Å of distance (Fig 3B). In the 3-fold center, the Terbium ion was successfully refined with 100% occupancy and it was tetrahedrally coordinated to OE1-Glu134 of the three subunits (at 2.2–2.4 Å distance) (Fig 3C). In each 4-fold channel, one water molecule was positioned and successfully refined as well as in the native protein.

With the aim to assess the 24-mer assembly of HFt-LBT and the degree of homogeneity of the sample in solution in a near native condition, HFt-LBT Tb(III) was analysed by cryo-electron microscopy.

**Fig 4:**
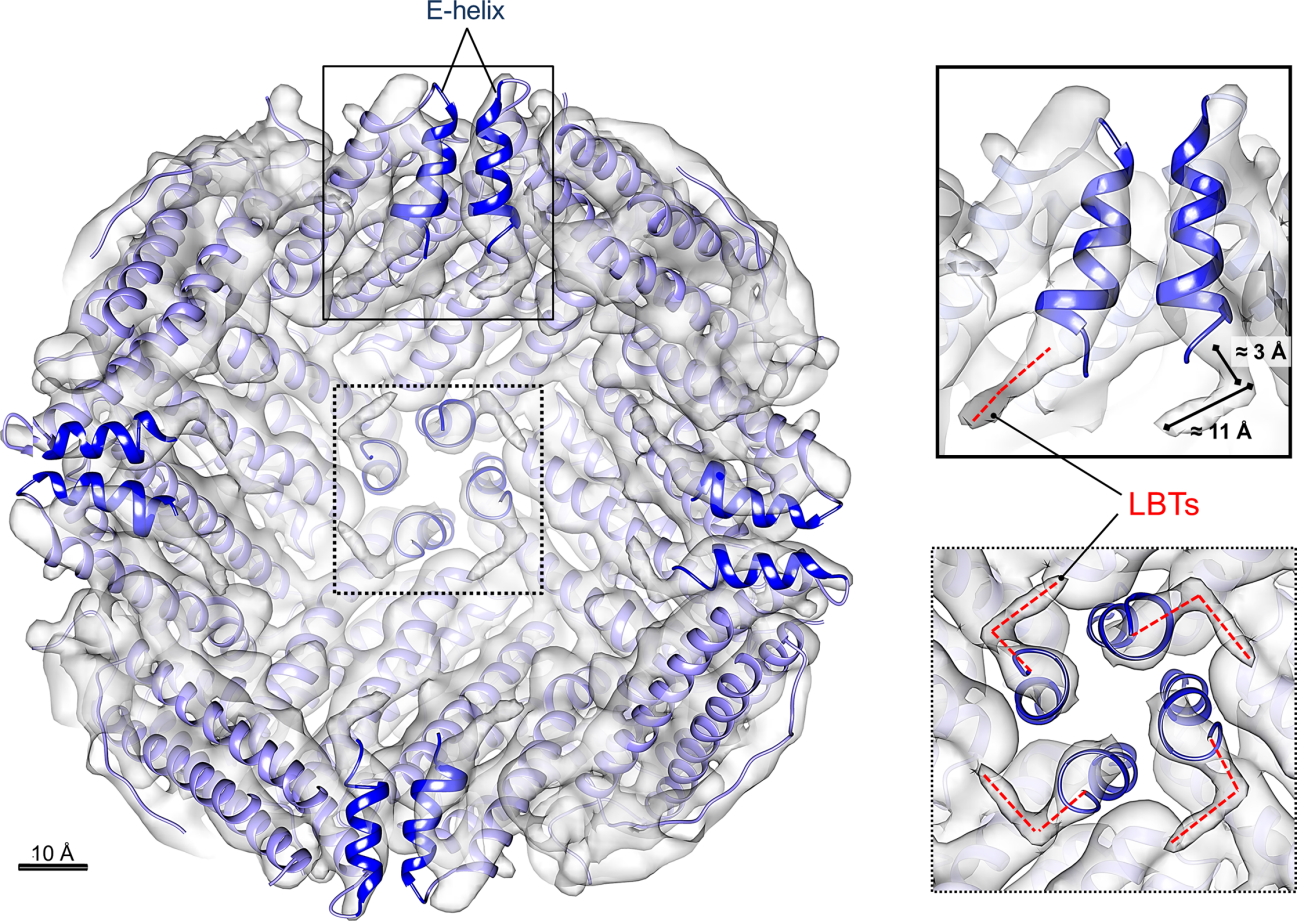
Cryo-electron microscopy structure of HFt-LBT. Internal view, sliced at the protein center, of the density map of HFt-LBT Tb(III) (resolution = 7.1 Å). The crystallographic model of HFt-LBT Tb(III) (purple ribbons) was rigid-body fitted into the Cryo-EM map (gray solid) using USCF Chimera [50]. The E helices at the C-terminal part of each monomer are highlighted in blue. LBT tails fused to the E helices are enlarged on the right panels showing the top view (dashed line box) and the side-view (continuous line box). Well-defined electron density extends over the end of the X-ray model as due to the presence of the terminal LBT tails and it approximately covers 14 Å distance, that corresponds to the length of 6–7 amino acids (main chain). Scale bar = 10 Å.

A qualitative analysis of the collected micrographs showed the high level of homogeneity and monodispersion of the HFt-LBT sample (S8 Fig). The processed imaged particles yielded a ≈7 Å resolution 3D map of the protein that overall corresponds to the high-resolution structure obtained by X-ray crystallography, indicating a perfect matching of the subunit assembly and the helix axes (Fig 4).

Nevertheless, differently from the X-ray map, the cryo-EM structure highlights the presence of significant L-shaped electron densities that protrude from the last C-term residue of the E helix of the X-ray HFt-LBT Tb(III) model to the internal cavity of the protein and that can be attributed to the lanthanide binding tags. These fragments, covering a distance of about 14 Å, correspond to segments of 6–7 residues each; they can also be seen in selected 3D classes (S4 Fig).

Thus, cryo-EM data clearly indicate that the E-helix is in its “flip” conformation in solution and presence of possible “flop” conformation, with the E-helix oriented outside the cavity, is not observed in any of the 3D classes [52]. Nevertheless, the considerable heterogeneity of the observed signals did not allow a reliable model reconstruction and only a partial (i.e. 6–7 aminoacids on a total of 17) segment of the tail was identified.

### Ferritins uptake by tumor cell lines

**Fig 5:**
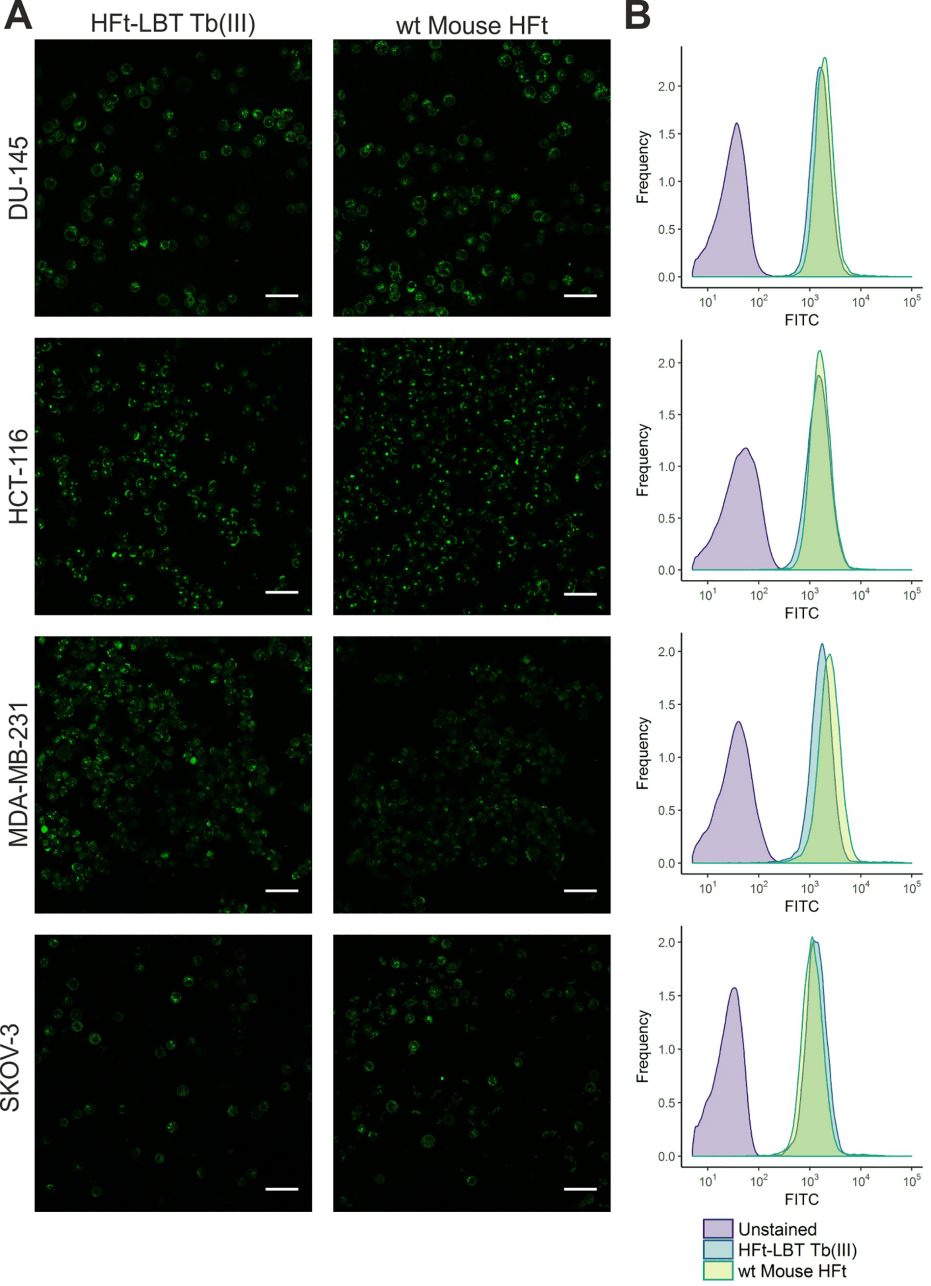
Confocal microscopy images and flow cytometry analysis of HFt-LBT Tb(III) uptake by selected tumor cell lines. DU-145, HCT-116, MDA-MB-231 and SKOV-3 cancer cells were incubated with either HFt-LBT Tb(III) or wild type mouse HFt (0.5 mg/ml) for 60 min. A) Images acquired by confocal microscopy showing side by side comparison of cellular distribution of HFt-LBT Tb(III) and HFt conjugated with FITC. Scale bar = 50 μm. B) Flow cytometry analysis of HFt-LBT Tb(III) and HFt cellular uptake.

After the demonstration that HFt-LBT Tb(III) maintained the overall structure of the wild type mouse HFt, binding and internalization of the construct was analyzed in selected cancer cell lines: in fact, it is known that HFt is recognized and internalized by CD71 receptor (also known as TfR1), which is overexpressed in many types of tumor cells but not in normal cells and healthy tissues [53]. Experiments on cells treated with the same amount (23.5 μM monomer, corresponding to 0.5 mg/ml) of HFt-LBT Tb(III) or wild type HFt were undertaken in order to study uptake efficiency by cancer cells by flow cytometry and confocal microscopy. As a baseline for FITC fluorescence, control cells untreated with FITC-ferritins were used. In order to exclude any signal generated from outside particles sticking on the cell membrane (due to unspecific binding or remaining from the washing steps), Trypan blue quenching was performed before FACS acquisition. In S9 Fig, FACS acquisition plots analysis is summarized, shown as the percentage of cells internalizing the nanoparticles: these data highlighted that HFt-LBT Tb(III) nanoparticles are efficiently taken up by all cells of each cell line. Confocal representative images of entire field of view of live cells incubated with HFt-LBT Tb(III) are shown in Fig 5A. Images confirmed the high extent of both HFt-LBT Tb(III) and wild type mouse HFt internalization and highlighted similar cellular distribution in the cytoplasm and in the perinuclear space.

## Discussion

In the present work, an engineered ferritin construct, bearing a lanthanide binding tag on the C-terminal end of each subunit of mouse H ferritin, was designed in order to build a biomolecular nano-system endowed with strong FRET sensitization properties. The peptide tag (LBT) was designed according to the findings of Martin *et al*. [24] that demonstrated efficient lanthanide chelating properties of a peptide sequence derived from Ca^2+^ binding sites from Troponin C EF hand motif. The LBT has six metal-binding residues that form a coordination sphere around a lanthanide (III) ion and is characterized by high affinity and strong FRET effect from the tag encoded tryptophan residue to the Terbium or Europium ions. Spectroscopic and crystallographic studies showed that the inner coordination sphere of Tb(III) bound to this sequence was free from water molecules [23], a key feature for luminescence experiments, given the quenching effect of O-H vibration [54]. Accordingly, the HFt-LBT construct exhibited high affinity Tb(III) binding as demonstrated by fluorescence spectroscopy measurements in solution. In these measurements, excitation of the tryptophan residue present in the center of the LBT provided an excellent FRET exchange to the Terbium atom with a sustained narrow emission at the 544 nm line typical of Terbium excited state decay (Fig 1). The thermodynamics of the Terbium binding process was however complex due to the well-known presence of additional metal binding sites to the ferritin 24-mer [30–32]. In particular, the observed fluorescence signal accounted for 1.7 Tb(III) ions per subunit indicates that the presence of the tryptophan residue within the tag is able to act as an antenna system not only for the Tb(III) ion bound within the LBT tag but also for a number of extra Tb(III) atoms bound to the natural ferritin binding sites. Control experiments carried out by titrating wild type mouse HFt with Tb(III) demonstrated negligible fluorescence contributions in the Tb(III) emission regions (S1 Fig). Such finding, in agreement with previous reports [30–31] and with current X-ray crystallography findings, confirms that Tb(III) is bound to the canonical iron binding sites in the threefold channels and ferroxidase sites but is only capable of receiving extremely weak energy transfer from the only Trp residue (namely, W94) present in the wild type protein.

Analysis of the three-dimensional structure of the HFt-LBT Tb(III) complex both by X-ray crystallography and cryo-EM demonstrated that the presence of the C-terminal tag does not affect the overall assembly of the protein and that the genetically fused tags point to the interior cavity. The peptide arm connecting the lanthanide binding loop to the C-terminal sequence is, as expected, flexible around the GSG spacer peptide connecting the C-terminal end to the LBT sequence, and does not allow for a complete resolution of the local structure [55]. Interestingly, cryo-EM 3D map reconstruction allowed the identification of low resolution but definite L-shaped density patterns relative to the first few (i.e. 6–7 on a total of 17) aminoacids of the tag. However, the possible multiple orientations of the loop region precluded the observation of the Tb(III) complex. X-ray data further demonstrated that Terbium ion is efficiently complexed at the threefold axis channels (8 channels per 24-mer) by side chains of E131 and D134 aminoacids from each of the three adjacent subunits. Further Tb(III) binding occurs stoichiometrically at the ferroxidase site (24 sites per 24-mer), coordinated by carboxyl residues E27 and E62 with minor participation of H65. In contrast, LBT bound Tb(III) atoms were not visible neither by X-ray diffraction nor by cryo-EM analysis. In summary, HFt-LBT construct is capable of: *i)* high affinity binding of 24 Tb(III) atoms, one per each lanthanide binding tag, *ii)* intermediate affinity binding of 20 Tb(III) atoms at the ferroxidase binding site (out of 24 available, as demonstrated by 75% occupancy in X-ray structure), and *iii)* lower affinity binding of 8 Tb(III) atoms at the entrance of the threefold channels. Hence, within the whole 24-mer a total of 56 Terbium atoms can be hosted, if we consider full occupancy of the ferroxidase sites. A full thermodynamic description concerning the dissection of the Tb sites thus appears extremely complex at this stage and will be evaluated in future on selected mutants. In particular, single and double mutations on the ferroxidase site and/or on the residues lining the threefold channels key residues will be needed. In the present investigation on confocal microscope, however, attempts to image live cells by direct excitation at 290–375 nm of the tryptophan residue in the lanthanide binding loop after ferritin uptake yielded very poor results due to the substantial fluorescence background originating from the pool of cytoplasmic protein, within the typical Terbium emission interval. Clearly, a time resolved fluorescence microscopy approach or a red shifted antenna system will be needed to expand the scope of the present work. We thus reported measurements in which HFt-LBT Tb(III) or wild type mouse HFt have been labeled with common FITC. Results demonstrate that the HFt-LBT Tb(III) complex is very efficiently uptaken by all four human tumor cell lines selected, in agreement with the identification of CD71 as the receptor of ferritin in humans [56]. In particular, DU145 (from a central nervous system metastasis, of primary prostate adenocarcinoma origin), MDA-MB-231 (from invasive ductal carcinoma), Hepatoma cell line and SK-OV-3 (highly resistant ovarian cancer cell line) were demonstrated for the first time to display a high uptake of both wild type mouseHFt and HFt-LBT Tb(III): this fact is remarkable, since these cancer cell lines overexpress CD71 receptor and are subject of cancer therapy studies focused on this receptor [54–60].

## Conclusions

In conclusion, we have shown that a ferritin nanocage can be engineered by addition of appropriate metal binding tags inside the cavity in order to provide specific additional metal sites in topologically selected positions. In this framework, the key physical property of the tag, namely chelating antenna system for Terbium, can be coupled to the CD71 receptor recognition properties of the ferritin protein within a construct that can be rationally designed and manipulated. We propose this approach as an alternative to the *quasi* random metal clusters (or small organic fluorophore) insertion into the ferritin cavity that has been commonly used by free diffusion of metal ions (or organic molecules) through the open pores on the surface of the macromolecule or by disassembly-reassembly of the 24-mer structure (“encapsulation” procedure) [8]. The importance of a guided allocation of metal sites inside the cavity is considered essential in order to proceed with rational positioning of antenna systems close to lanthanide sites with the aim of designing the best geometry for efficient FRET. Rational design of such metal binding sites would foster more advanced applications such as the construction of up-converting nanoparticles or ultra-bright fluorescent organic polymers for single molecule detection. The present construct thus represents a first step towards the engineering of the ferritin cavity in order to generate a more efficient fluorescence/luminescence intracellular tracking system according to a structurally guided, rational design approach.

## Supporting information

S1 Fig SEC profile of HFt-LBT. Molecular weight determination by gel filtration was carried out by comparing the elution volume of HFt-LBT with the values obtained for several known calibration standards. HFt-LBT, loaded onto a HiLoad 26/600 Superdex 200 column, elutes as a single pick at 70 ml, the elution volume expected for the 24meric state of the protein, preceded by two small peaks corresponding to higher molecular weight aggregates (58 and 48 min). The inset shows the native page of HFt-LBT (lane 2) and HFt-LBT treated with of 1 mM TCEP (lane 3). Small amount of dimeric (dimers of 24-mers) or trimeric (trimer of 24-mers) can be detected both in the SEC profiles and in the corresponding native page. The higher MW multimers have been attributed to the presence of intermolecular disulfide bridges, as their presence is greatly decreased by addition of TCEP.
(TIF)

S2 Fig SDS-PAGE of purified HFt-LBT. Line 1: Precision Plus Protein Unstained Protein Standards (BioRad); line 2: purified HFt-LBT.
(TIF)

S3 Fig MALDI-TOF mass spectrum of HFt LBT. The identified molecular mass of 22531 Da corresponds to theoretical protein mass (22662 Da) subtracted of the contribution of a methionine (131 Da).
(TIF)

S4 Fig Representative 3D classes averages. Classes 3 and 5 contain noisy contributions; other classes are estimated high quality for 3D reconstructions obtained with RELION 2.0. Even at such low resolution, it is possible to notice non trivial structures inside the cavity.
(TIF)

S5 Fig Fourier shell correlation (FSC) of the final 3D map reconstruction (see Fig 4). The resolution calculation is based on the ‘gold-standard’ protocol that assures the independence of the half-set reconstructions. Dashed purple line: FSC = 0.143. Final resolution: 7.1 Å.
(TIF)

S6 Fig X-ray emission spectrum of HFt-LBT-Tb. L emission lines of terbium at 7.5, 8.2 and 8.5 keV are displayed. Additional lines at 6.3 and 7.0 keV are ascribed to iron ions.
(TIF)

S7 Fig Anomalous difference map peaks of terbium. The structure is represented as cartoon, the residues surrounding the terbium ion are represented as sticks, terbium is represented as spheres and the map, contoured at 3 σ, is represented as a blue mesh. A) Terbium at the ferroxidase center B) Terbium at the 3-fold axes.
(TIF)

S8 Fig Sample micrograph of HFt-LBT Tb(III). The sample was highly homogeneous and monodisperse. Scale bar = 100 nm.
(TIF)

S9 Fig Representative FACS images: FITC labelled HFt-LBT Tb(III) and wt mouse HFt were loaded within the four cell lines: HCT, MDA-MB, DU-145 and S-KOV3. V500: violet-excitable dye engineered to improve brightness and reduce spectral overlap into the FITC channel.
(TIF)

S10 Fig Effect of iron II addition to HFt-LBT Tb(III) adduct. Fluorescence spectra of HFt-LBT Tb(III) (red line) and HFt-LBT Tb(III) containing 10 μM ammonium iron(II) sulfate hexahydrate (blue line). Samples were at the same protein concentration (1 μM monomer). Spectra in the presence of iron were recorded after 2, 4, 8, 16 and 24 h at 25°C in 0.1 M MES buffer pH 6.4 and did not show detectable time dependent changes. For clarity, only the spectrum after 24 h is shown. Addition of higher amount of iron resulted in hazyness and precipitation, most likely due to the formation of insoluble iron hydroxide precipitates.
(TIF)

S1 Table Data collection and refinement statistics. (PDF)
