# Supplementary figures and images for: Engineered ferritin for lanthanide binding
Source: PLoS One. 2018 Aug 13;13(8):e0201859. doi: 10.1371/journal.pone.0201859 (PMC6089422; doi:10.1371/journal.pone.0201859)

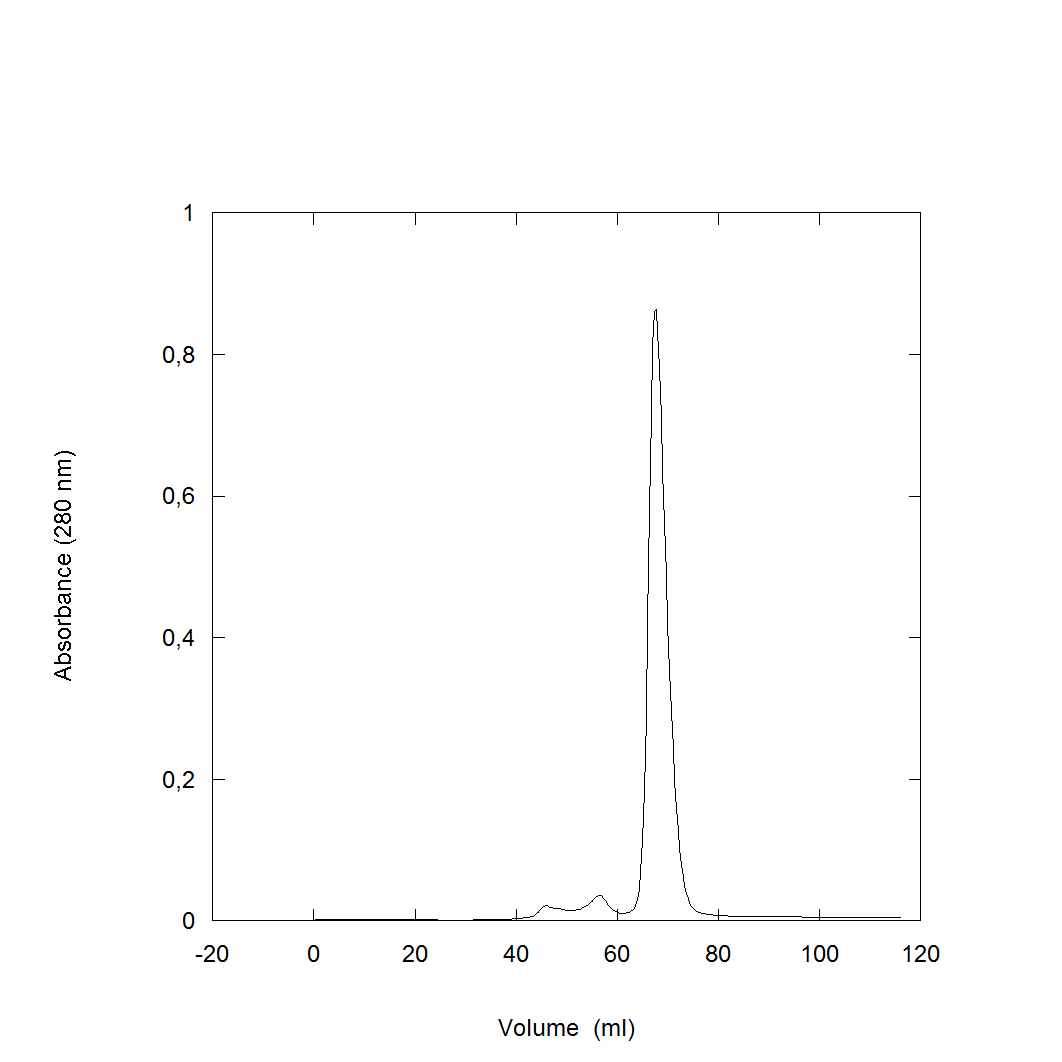

Supplement: S1 Fig — Molecular weight determination by gel filtration was carried out by comparing the elution volume of HFt-LBT with the values obtained for several known calibration standards. HFt-LBT, loaded onto a HiLoad 26/600 Superdex 200 column, elutes as a single pick at 70 ml, the elution volume expected for the 24meric state of the protein, preceded by two small peaks corresponding to higher molecular weight aggregates (58 and 48 min). The inset shows the native page of HFt-LBT (lane 2) and HFt-LBT treated with of 1 mM TCEP (lane 3). Small amount of dimeric (dimers of 24-mers) or trimeric (trimer of 24-mers) can be detected both in the SEC profiles and in the corresponding native page. The higher MW multimers have been attributed to the presence of intermolecular disulfide bridges, as their presence is greatly decreased by addition of TCEP. (TIF) [file pone.0201859.s001.tif]

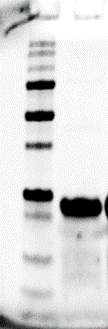

Supplement: S2 Fig — Line 1: Precision Plus Protein Unstained Protein Standards (BioRad); line 2: purified HFt-LBT. (TIF) [file pone.0201859.s002.tif]

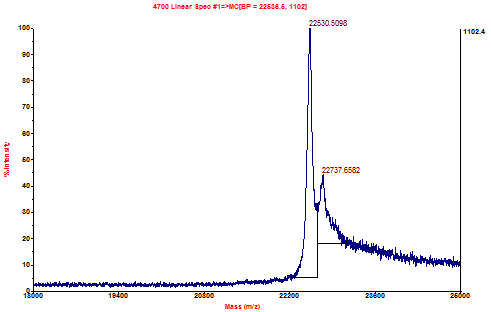

Supplement: S3 Fig — The identified molecular mass of 22531 Da corresponds to theoretical protein mass (22662 Da) subtracted of the contribution of a methionine (131 Da). (TIF) [file pone.0201859.s003.tif]

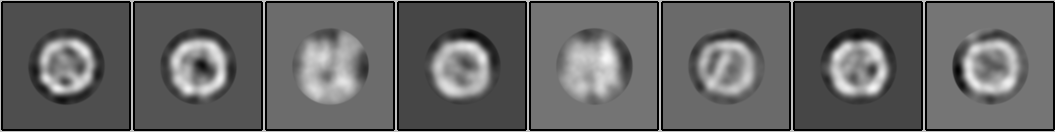

Supplement: S4 Fig — Classes 3 and 5 contain noisy contributions; other classes are estimated high quality for 3D reconstructions obtained with RELION 2.0. Even at such low resolution, it is possible to notice non trivial structures inside the cavity. (TIF) [file pone.0201859.s004.tif]

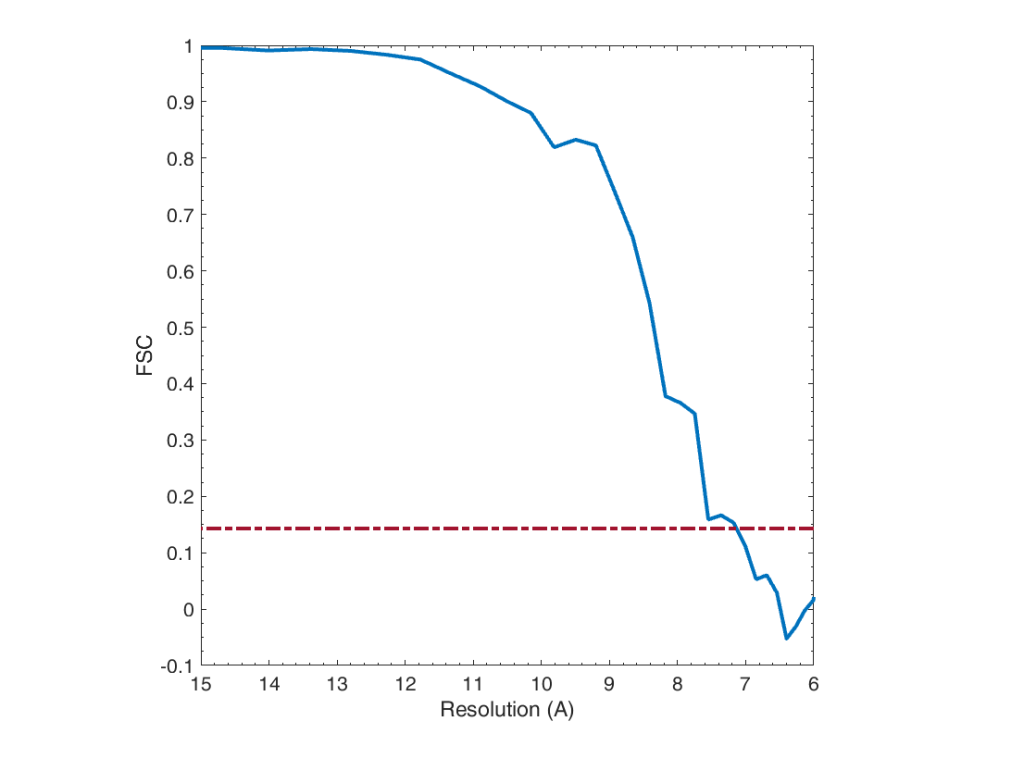

Supplement: S5 Fig — The resolution calculation is based on the ‘gold-standard’ protocol that assures the independence of the half-set reconstructions. Dashed purple line: FSC = 0.143. Final resolution: 7.1 Å. (TIF) [file pone.0201859.s005.tif]

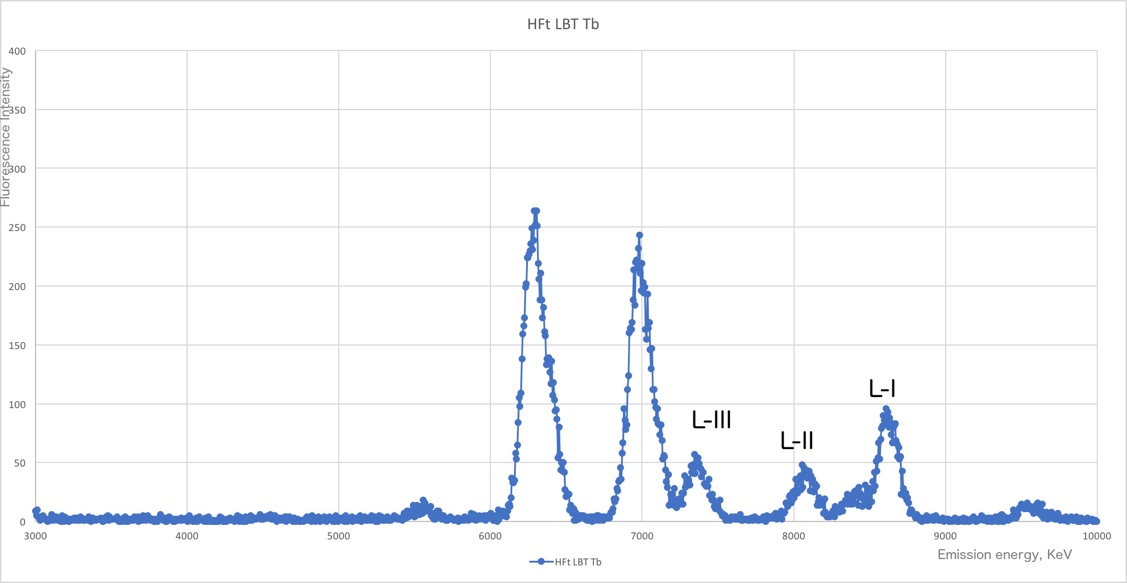

Supplement: S6 Fig — L emission lines of terbium at 7.5, 8.2 and 8.5 keV are displayed. Additional lines at 6.3 and 7.0 keV are ascribed to iron ions. (TIF) [file pone.0201859.s006.tif]

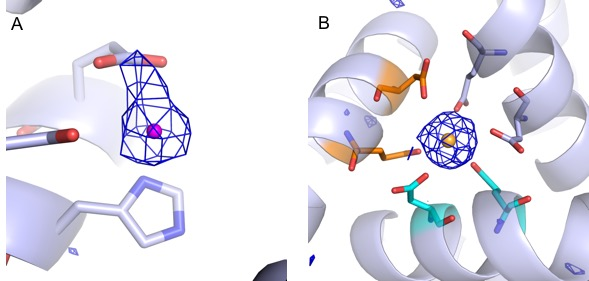

Supplement: S7 Fig — The structure is represented as cartoon, the residues surrounding the terbium ion are represented as sticks, terbium is represented as spheres and the map, contoured at 3 σ, is represented as a blue mesh. A) Terbium at the ferroxidase center B) Terbium at the 3-fold axes. (TIF) [file pone.0201859.s007.tif]

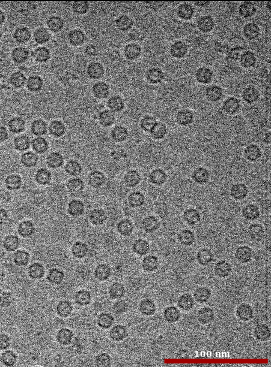

Supplement: S8 Fig — The sample was highly homogeneous and monodisperse. Scale bar = 100 nm. (TIF) [file pone.0201859.s008.tif]

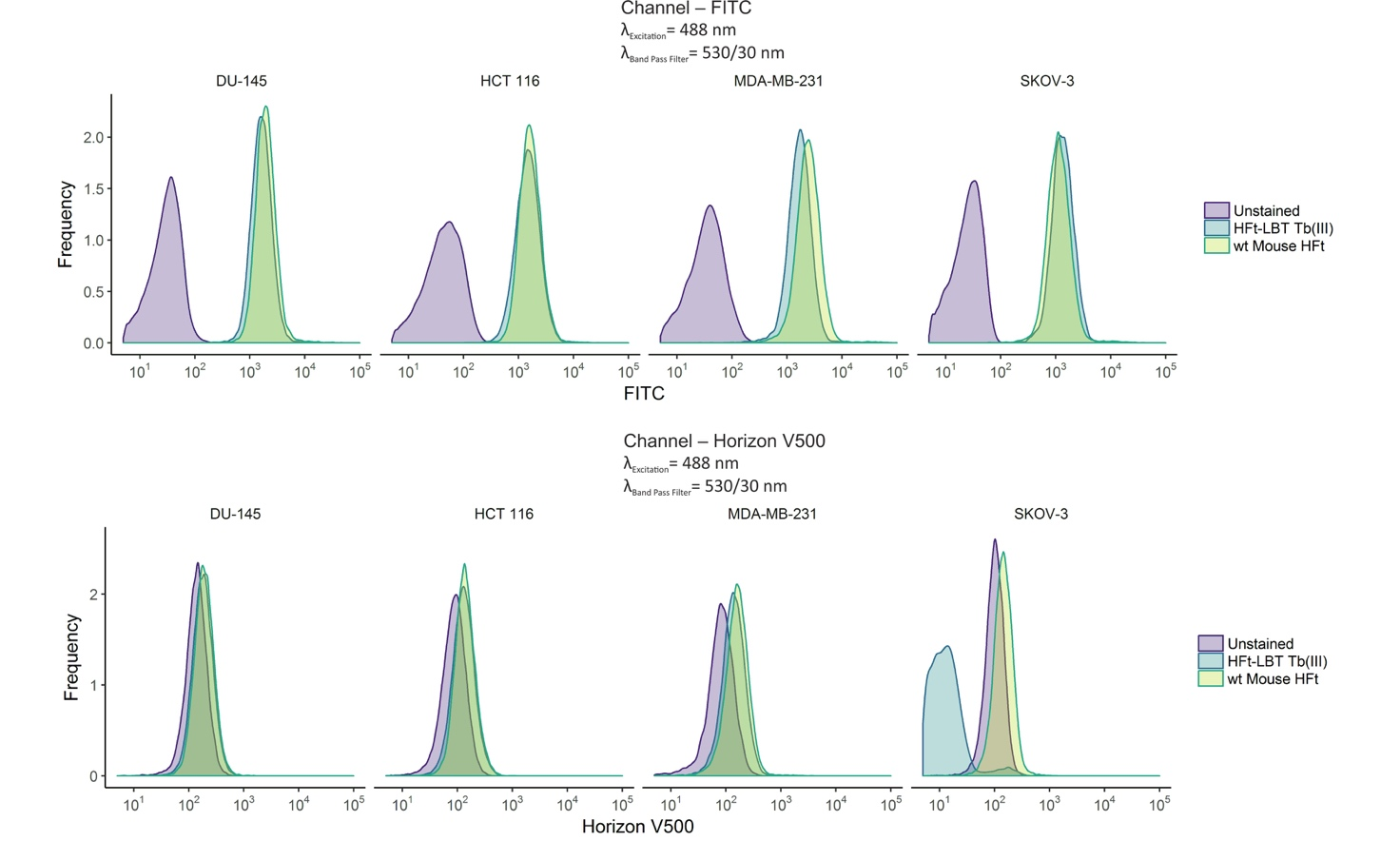

Supplement: S9 Fig — V500: violet-excitable dye engineered to improve brightness and reduce spectral overlap into the FITC channel. (TIF) [file pone.0201859.s009.tif]

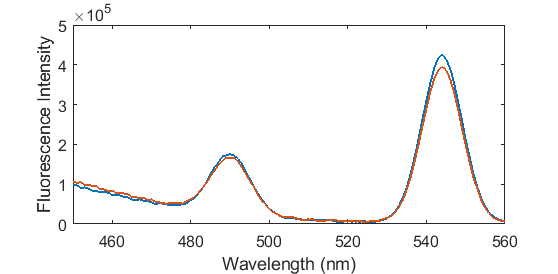

Supplement: S10 Fig — Fluorescence spectra of HFt-LBT Tb(III) (red line) and HFt-LBT Tb(III) containing 10 μM ammonium iron(II) sulfate hexahydrate (blue line). Samples were at the same protein concentration (1 μM monomer). Spectra in the presence of iron were recorded after 2, 4, 8, 16 and 24 h at 25°C in 0.1 M MES buffer pH 6.4 and did not show detectable time dependent changes. For clarity, only the spectrum after 24 h is shown. Addition of higher amount of iron resulted in hazyness and precipitation, most likely due to the formation of insoluble iron hydroxide precipitates. (TIF) [file pone.0201859.s010.tif]
