# Supplementary material for: Engineered ferritin for lanthanide binding
Source: PLoS One. 2018 Aug 13;13(8):e0201859. doi: 10.1371/journal.pone.0201859 (PMC6089422; doi:10.1371/journal.pone.0201859)
Supplement: S1 Table — (PDF) [file pone.0201859.s011.pdf]

| Data Collection | apoHFt_TBP | HFt_TBP_TB |
|---|---|---|
| Wavelength (Å) | 1.0 | 1.4 |
| Space group | I222 | I222 |
| Cell Dimensions | 237.31 237.47 237.53 90.00 90.00 90.00 | 237.25 237.56 237.57 90.00 90.00 90.00 |
| Resolution (Å) | 48.48-2.85 (2.9-2.85) | 48.48-2.65 (2.70-2.65) |
| $R_{merge}$ | 0.109 (0.833) | 0.133 (1.135) |
| I/σI | 17.6 (2.5) | 12.6(1.4) |
| Completeness (%) | 100 (100) | 99.2 (94.3) |
| Redundancy | 6.9 (6.8) | 6.7 (5.3) |
| **Refinement** | | |
| Resolution (Å) | 48.48-2.85 (2.9-2.85) | 48.48-2.65 (2.70-2.65) |
| No. unique reflections | 61020 (4475) | 191449 (8966) |
| $R_{work}$ | 0.137 (0.352) | 0.164 (0.266) |
| $R_{free}$ | 0.167 (0.374) | 0.176 (0.282) |
| Ligand/Ion | 32 Iron Ions | 32 Terbium Ions |
| Water | 12 | 11 |
| R.m.s. Deviations | | |
| Bond length (Å) | 0.004 | 0.004 |
| Angles (°) | 0.711 | 0.623 |
